# Competitive dynamics of arbuscular mycorrhizal fungi as depending on fungal traits and host plant species

**DOI:** 10.1007/s00572-026-01254-7

**Published:** 2026-02-24

**Authors:** Henry J. De La Cruz, Nicolás Marro, Milena Caccia, Kateřina Žďárská, Martina Janoušková

**Affiliations:** 1 Institute of Botany, The Czech Academy of Sciences, Zámek 1, Průhonice, 252 43 Czech Republic; 2 Department of Botany, Faculty of Science, Charles University, Benátská 2, Prague, 128 00 Czech Republic; 3 Instituto Multidisciplinario de Biología Vegetal (IMBIV), CONICET, Facultad de Ciencias Exactas, Físicas y Naturales, Universidad Nacional de Córdoba, Córdoba, Argentina

**Keywords:** AMF, Interactions, Competition, Fungal traits, Host plant species, Abundance

## Abstract

Arbuscular mycorrhizal fungi (AMF) are ubiquitous root-associated symbionts, but competitive interactions among coexisting taxa remain poorly understood. The variation in colonization and resource-acquisition strategies drive competition, shaping the relative abundances of AMF within their communities and mycorrhiza functioning. However, the factors that determine the competitive outcomes have received limited experimental attention. We hypothesized that AMF abundances, competitive responses, and contributions to plant growth would vary according to fungal traits and host plant species. Three AMF isolates of contrasting growth and competitive abilities, each representing one AMF species, were mono- and co-inoculated under six host plant species. Fungal abundance, root colonization (RC), and mycorrhizal growth response (MGR) were measured at early and late stages of the symbiosis.

Fungal competitive responses depended on the isolate’s root colonization strategy in monoculture and mycorrhizal stage. The fast-colonizing isolate dominated initially, while slower-growing isolates displayed different temporal patterns, either declining or maintaining their abundance over time. Our findings suggest competitive outcomes among AMF that were asymmetric at early stages but became more symmetric later. The host plant species importantly modulated the dominance of the fast root-colonizer. MGR was positively associated with total AMF abundance and RC, yet the most abundant isolate did not necessarily provide the greatest growth benefits to the host plant. Our results suggest that maintaining a diversity of AMF functional types, rather than introducing a single highly competitive isolate, is favourable to the establishment of stable and efficient plant-AMF associations.

**Supplementary Information:**

The online version contains supplementary material available at 10.1007/s00572-026-01254-7.

## Introduction

Among soil microorganisms, arbuscular mycorrhizal fungi (AMF) are the most widespread root symbionts of plants, establishing associations with more than 80% of terrestrial plants (Smith and Read 2010). In exchange for carbon supplied by the host, AMF confer multiple benefits to plants, including improved acquisition of soil nutrients and increased tolerance to both abiotic and biotic stresses (e.g., Smith et al. 2003; Delavaux et al. 2017). Although the positive effects of AMF have been extensively documented (e.g., Branco et al. 2022; Marro et al. 2022), interactions among coexisting AMF taxa and how they influence one another remain poorly explored. These interactions may encompass a range of outcomes, from competition to neutrality or facilitation, depending on ecological context (Eisenhauer 2012; Thonar et al. 2014). AMF taxa differ in their ability to colonize plant roots and acquire resources (Hart et al. 2001; Chagnon et al. 2013; Horsch et al. 2023; Antunes et al. 2025), which may promote competitive interactions that determine the relative abundances of fungal species within AMF communities. A shift in the AMF abundances can, in turn, alter nutrient availability and plant growth (Veresoglou et al. 2012; Werner and Kiers 2015; Blažková et al. 2021), impacting overall on plant community structure (van Der Heijden et al. 1998; Hart et al. 2003) and ecosystem functioning (Powell and Rillig 2018).

The competition between AMF species can be influenced by various factors (Montesinos-Navarro et al. 2019; Yu and He 2022; Bennet and Bever 2009; Delavaux et al. 2017), including differences in their traits (Chagnon et al. 2013; Chaudhary et al. 2022). For example, AMF with fast-growing extraradical hyphae and extensive hyphal networks may be efficient in exploring resources, thereby outcompeting slower-growing fungi in nutrient acquisition (Hart et al. 2001; Hart and Reader 2002; Bennet and Groten 2022). Fungal traits related to rapid root colonization can be crucial for establishing a dominant presence during early stage of mycorrhiza development (Werner and Kiers 2015; Lekberg and Koide 2005; Voříšková et al. 2019). Similarly, AMF with faster spore germination are likely to be more successful in the early stages of their life cycles, establishing the symbiotic connection with the host faster and exploiting available resources before other, slower-growing species can (Horsch et al. 2023). In long-term interactions, in contrast, traits that contribute to the persistence and resilience of AMF, (e.g., higher spore longevity), can play a significant role in shaping the composition of mycorrhizal communities (Chagnon et al. 2013).

In natural ecosystems, individual plant roots are typically colonized by many different AMF species (Öpik et al. 2009). However, not all plant species form associations with every fungal species present in a community, resulting in non-random patterns of symbiont association (Montesinos-Navarro et al. 2012; Bever et al. 2009; Klironomos 2003). Some AMF are functionally more compatible with certain host plants (Sepp et al. 2019), which promote their proliferation within the root system and shape the local fungal community composition in terms of richness and abundance (Bever et al. 1996; van Der Heijden et al. 1998). When the preferentially associated AMF are also more effective partners, i.e., more efficient in nutrient acquisition, carbon exchange, or stress mitigation, these associations can enhance plant growth and performance (Eom et al. 2000; Helgason et al. 2002; Hoeksema et al. 2010). The latter can also lead to stronger symbiotic relationships and increased competitive ability for both the fungus and the plant (Knegt et al. 2016). If fungal dominance reflects functional efficiency within the symbiosis, the most competitive or abundant AMF would be expected to contribute most to host mycorrhizal benefits (Hoeksema et al. 2010; Bever et al. 2009; Kiers et al. 2011; Chagnon et al. 2013).

Despite important insights into environmental drivers of AMF community composition, there is still little information about competition among AMF species and potential modulating factors. Following early pioneer studies (Abbott and Robson 1984a, b; Wilson 1984; Wilson and Trinick 1983; Pearson et al. 1993, 1994), only a few experimental studies have directly evaluated competition among AMF. Most of these studies have focused on the identity and relative abundance of co-inoculated fungi, often using a single host species (Thonar et al. 2014; Engelmoer et al. 2014; Blažková et al. 2021; Yu and He 2022) or, at most, two host species (Jansa et al. 2008). Consequently, it remains unclear to what extent AMF community establishment is driven by inherent fungal traits related to interspecific competition, and how this may be modulated by host plant preferences for a particular fungal species. Understanding these competitive dynamics is essential for managing root-associated AMF communities and predicting their success in ecological restoration and agroecosystems (van Der Heijden et al. 2015; Bennett and Groten 2022).

In this study, variability of competitive interactions among AMF species in different host plant species was evaluated by tracking the abundance of each taxon using DNA-based quantification in two stages of mycorrhizal development (early and late). To this end, three isolates of common co-occurrence were selected to represent one fast-growing AMF and strong competitor, and two less competitive AMF of similar growth rates, based on previous studies and observations (Supplementary Table S1). We hypothesized that the relative abundances of the fungal isolates and their responses to competition in co-inoculation would reflect their abundances in mono-inoculation, based on the assumption that intrinsic fungal traits determine outcomes when fungi are grown together. We further hypothesized that ratios of AMF abundances would vary among host plant species, based on the established knowledge that host identity influences AMF community composition (Eom et al. 2000; Lekberg and Waller 2016). Finally, we hypothesized that the most abundant and competitive AMF would contribute most to the mycorrhizal benefits of the host plant assuming that dominant taxa provide the greatest contribution to plant growth or nutrient uptake, while functional complementarity among co-occurring taxa may also play a role.

## Materials and methods

### Fungal and plant species

Three AMF isolates, belonging to different species, were used in this study: *Rhizophagus irregularis* (Błaszk., Wubet, Renker & Buscot) Walker & Schüßler isolate PH5, *Entrophospora claroidea* (N.C. Schenck & G.S. Sm.) Błaszk., Niezgoda, B.T. Goto & Magurno isolate BEG23 (formerly known as *Glomus claroideum* or *Claroideoglomus claroideum*), and *Funneliformis mosseae* (Nicolson & Gerd.) Walker & Schüßler isolate BEG95. The *R. irregularis* isolate was originally obtained from a heavy metal contaminated grassland site in the Czech Republic (Rydlová and Vosátka 2003) while *F. mosseae* and *E. claroidea* isolates are registered in The International Bank of the Glomeromycota (https://www.ibeg.eu/). In previous studies, these three fungal isolates established communities dominated by the fastest root-colonizer *R. irregularis* and with *E. claroidea* and *F. mosseae* in small and similar relative abundances (Janoušková et al. 2013; Voříšková et al. 2019; Blažková et al. 2021). The inoculum of each fungal isolate was produced with *Desmodium sp.* as host plant, grown in 2 L pots in a sand-zeolite mixture (1:1, v: v). Pots were harvested after six months, the substrate air-dried and homogenized, and roots cut to fragments of about 5 mm. To verify the purity and quality of the inoculum, a subsample was wet-sieved and checked by stereomicroscope to confirm abundant sporulation. Inoculum was standardized through a uniform preparation protocol, assuming that all isolates had reached a comparable plateau level of infectivity (Voříšková et al. 2019). Finally, the air-dried substrate from the cultures was stored at 3–4 °C in a refrigerator until use, i.e., for c. two weeks.

The study was conducted with six host plant species: *Plantago lanceolata* L. (Plantaginaceae), *Centaurea scabiosa* L. (Asteraceae), *Galium verum* L. (Rubiaceae), *Briza media* L. (Poaceae), *Sanguisorba minor* Scop. (Rosaceae), and *Thymus pulegioides* L. (Lamiaceae). These species are common in dry, nutrient-poor grasslands and differ in their position within the C-S-R Grime triangle as well as in mycorrhizal responsiveness (see Supplementary Table S2). Seeds of all host species were obtained commercially from Planta Naturalis (Czech Republic).

## Experimental design

**Fig. 1:**
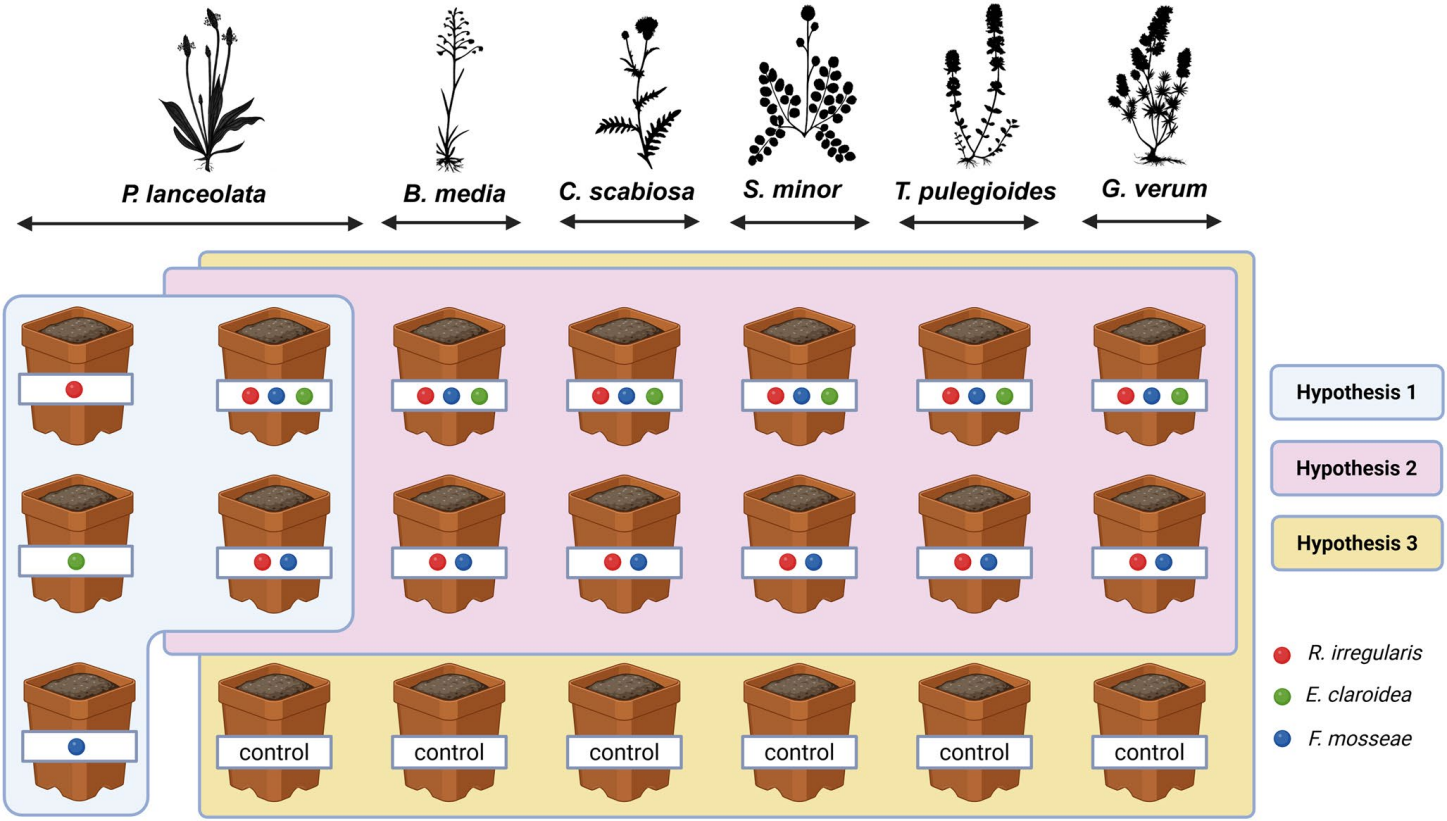
Schema of the greenhouse experiment, each pot represents one experimental treatment, each colour dot the inoculum of an arbuscular mycorrhizal fungal isolate, as specified in the legend. ‘Control’ means non-inoculated pots. The treatments used to address each hypothesis are indicated with different colours in the background. Each treatment was established in 12 replicates to harvest half of them during the early stage and the other half during the late stage of mycorrhizal development

Interspecific competition among the fungal isolates was assessed by growing *R. irregularis*, *F. mosseae* and *E. claroidea* either individually (mono-inoculation) or together (co-inoculation of all three isolates, or only two isolates: *F. mosseae* and *E. claroidea*) using *P. lanceolata* as the model host (Fig. 1, blue-shaded area). The influence of host plant species on AMF abundance was evaluated by applying the two co-inoculation treatments to five additional plant species (Fig. 1, pink-shaded area). Non-inoculated individuals of all six host species were included as non-mycorrhizal controls to examine the relationship between AMF abundance and mycorrhizal benefits of the host plant (Fig. 1, yellow-shaded area). Each inoculation and control treatment was replicated twelve times, with six replicates harvested at five weeks (early stage) and six at twenty-three weeks (late stage). Harvest times were chosen to contrast early establishment with mature stages of the symbiosis, rather than to define a specific temporal threshold for competitive dynamics.

The experiment was conducted from July to December 2023 in a greenhouse with no modifications of environmental conditions. From October onward, heating (night/day temperatures of 15–35 °C) and supplemental lighting (12-h photoperiod) were provided using high-power LED panels (EuledK 200HS; Euled s.r.o., Czech Republic). The plants were grown in 1 L plastic pots in a sand-zeolite mixture (1:1, v: v), sterilized by autoclaving (2 × 30 min. at 120 °C). As summarized in Supplementary Table S4, the plastic pots were first filled with 200 mL of the sterile substrate, then with a treatment-specific “inoculum layer” (as specified below), and with 200 mL of the sterile substrate on the top. Dried inoculum of the AMF isolates was mixed with the sterilized substrate to prepare 600 mL of the inoculum layer. Within this volume, the inoculum of each isolate was added in the amount of 2.5% (v/v) independently of whether it was inoculated alone or co-inoculated. The non-inoculated controls received blank inoculum sterilized by autoclaving. In order to equalize the addition of organic matter to all treatments, the blank inoculum was added in the amount of 7.5% to the inoculum layer of the control treatment, and also in the amount of 5% to the mono-inoculated treatments (that received 2.5% of living inoculum) and 2.5% to the treatments inoculated with two isolates (that received 5% of living inoculum). Additionally, each replicate pot received 10 ml of bacterial filtrate to equalise the microbial community in all pots. The filtrate was prepared from a mixture of all the cultures used for inoculation by passing soil suspension twice through a filter paper (Whatman No. 1, pore size: 11 μm). During the cultivation, plants were supplied with the modified White’s nutrient solution P2N3 described by Gryndler et al. (1992), 50 mL per week.

### Data collection

At the harvest (both early and late stage), shoots of each plant were cut off and dried at 70 °C for three days and then weighed to obtain the shoot dry weight. Root systems were washed and dried with paper towels. Samples for molecular analyses and determination of root colonization were randomly collected from the upper two thirds of the root systems after cutting the roots to 1 cm fragments and homogenizing. Aliquots of 100 mg fresh weight of roots for molecular analyses were immediately frozen in liquid nitrogen and stored at -80 °C. Samples of about 1 g fresh weight of roots were cleared in 10% KOH and stained with 0.05% Trypan Blue in lactoglycerol (Koske and Gemma 1989) to estimate AMF colonization. Root colonization (RC), used as an index of total fungal abundance, was quantified by the magnified intersection method (McGonigle et al. 1990). For each sample, 100 intersections were scored across 30 root segments, and the percentage of root length colonized by hyphae, arbuscules, and vesicles was microscopically recorded. Frozen root samples were ground in liquid nitrogen and DNA was extracted using DNeasy Plant Mini kit (Qiagen, Hilden, Germany) according to the manufacturer instructions. The quality and quantity of the extracted DNA were assessed using a NanoDrop spectrophotometer (Thermo Fisher Scientific, Waltham, MA, USA). The abundance of each fungal isolate was determined by SYBR Green-based quantitative real-time PCR (qPCR) with isolate-specific primers (see Supplementary Table S3) on a LightCycler 480 II Real-Time PCR Instrument (Roche). The thermal cycling program was set up with initial pre-incubation at 95 °C for 10 min, 40 cycles of 95 °C for 10 s (denaturation), 58 °C for 30 s (annealing) and 72 °C for 15 s (elongation and data collection). rDNA copy numbers were calculated from standard curve regressions, generated from serial dilutions of purified amplicons as described by Voříšková et al. (2019), and normalized to total DNA concentration (copies ng^− 1^ DNA) to correct for differences in extraction yield among samples (Janoušková and Caklová 2020). qPCR estimates were interpreted as relative measures, since rDNA copy number variation and extraction efficiency can bias absolute abundance.

### Data analysis

The competitive response of each AMF isolate was quantified using the Relative Interaction Index (RII; Armas et al. 2004), calculated as (Bw – Bo) / (Bw + Bo), where Bw and Bo represent fungal isolate abundance in co- and mono-inoculation, respectively. Positive RII values indicate an increase in focal AMF abundance due to the presence of another AMF (i.e., facilitation), negative values indicate a decrease (i.e., competition), and values not different from zero indicate no effect (i.e., neutral interaction). The plant benefit from mycorrhiza was evaluated using the mycorrhizal growth response (MGR = log[M/NM]), where M is the shoot dry weight of a mycorrhizal plant replicate and NM is the mean shoot dry weight in the corresponding non-mycorrhizal treatment. Positive MGR values indicate growth promotion, and negative values indicate reduced performance.

The differences in AMF abundance among fungal isolates were analysed separately for the mono- and co-inoculation treatments using two-way ANOVAs, with fungal isolate and mycorrhizal stage as fixed factors. Fungal competitive responses were further evaluated with two-way ANOVAs for each co-inoculation treatment, using the RII as the response variable and fungal isolate and mycorrhizal stage as fixed factors.

The effects of host plant species on AMF abundances were assessed using three-way ANOVAs, with abundance as the response variable and host plant species, fungal isolate, and mycorrhizal stage as fixed factors. Relationships between AMF abundance and the MGR were analysed using linear models fitted separately for each co-inoculation treatment and mycorrhizal stage. MGR was specified as the response variable, while fungal abundance (*R. irregularis*, *F. mosseae*, *E. claroidea)* and host plant species were included as predictors. Partial R² values were calculated to estimate the relative contribution of each predictor. Furthermore, the relationships of MGR with RC were determined using linear models fitted across all plant species, separately for each mycorrhizal stage.

All ANOVAs were followed by Tukey’s HSD tests (*p* < 0.05). Student’s t-tests were additionally applied to determine whether individual RII and MGR values differed significantly from zero. Model assumptions of normality and homoscedasticity were verified by inspection of residual plots (Q-Q plot and histograms) as well as Shapiro-Wilk test, and multicollinearity among predictors was assessed using variance inflation factors (VIF). All analyses were performed in R v4.2.2 (R Core Team, 2022).

## Results

### Abundances and competitive responses of the three AMF in *P. lanceolata*

**Fig. 2:**
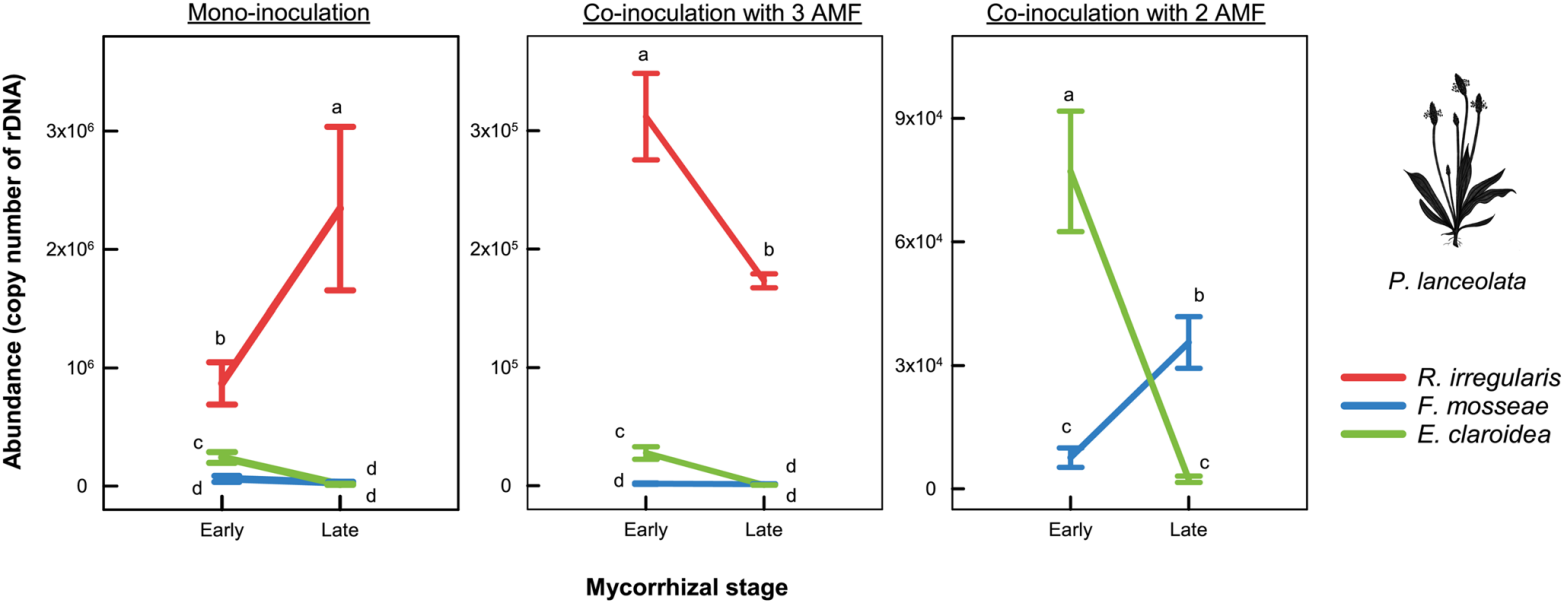
Abundances (as copy numbers of nuclear ribosomal DNA ng^− 1^ root-extracted DNA) of arbuscular mycorrhizal fungi (AMF: *R. irregularis*, *F. mosseae*, and *E. claroidea*) in the root system of *Plantago lanceolata*, during two mycorrhizal stages (early and late): mono-inoculation (each AMF cultivated separately), co-inoculation with 3 AMF and co-inoculation with 2 AMF. Each point is mean (*n* = 6) with standard error displayed by vertical lines. Different letters denote significant differences between the means within each partial figure (*a posteriori Tukey test*, *p < 0.05*)

Significant interactions of the factors mycorrhizal stage and fungal isolate (see Supplementary Table S5) confirmed that differences between the abundances of the AMF depended on mycorrhizal stage in mono-inoculation (F_2, 27_ = 7.17, *p* < 0.01) and both co-inoculation treatments (3 AMF: F_2, 21_ = 37.29, *p* < 0.0001; 2 AMF: F_1, 20_ = 95.78, *p* < 0.0001). In mono-inoculation, *R. irregularis* was more abundant than *F. mosseae* and *E. claroidea* at both stages. *E. claroidea* was more abundant than *F. mosseae* at the early stage but both isolates had similar abundance at the late stage. The pattern was similar when all three AMF were co-inoculated, except that the abundance of *R. irregularis* decreased between the early and late stage in co-inoculation but increased in mono-inoculation. In the dual inoculation, *E. claroidea* had higher abundance than *F. mosseae* at the early stage but became less abundant at the late stage reflecting a decline of *E. claroidea* and an increase of *F. mosseae* over time (Fig. 2).

**Fig. 3:**
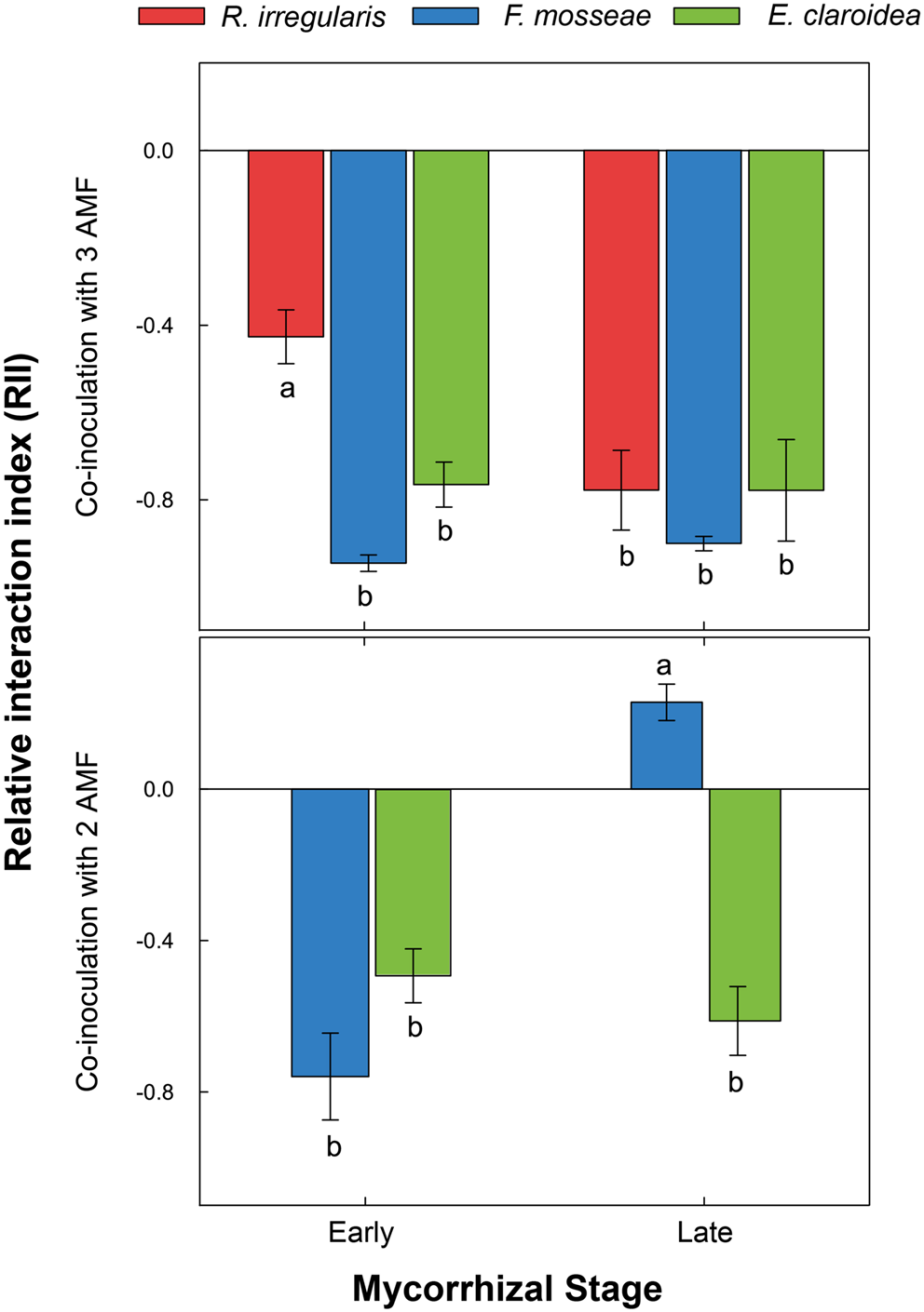
Competitive responses of arbuscular mycorrhizal fungi (AMF: *R. irregularis*, *F. mosseae*, and *E. claroidea*) in the root system of *P. lanceolata*. Relative Interaction Index (RII) values are shown per co-inoculation and stage. Each bar is mean (*n* = 6) with standard error displayed by vertical lines. Different letters denote significant differences between the fungal isolates (*a posteriori Tukey test*, *p < 0.05*). All values are significantly different from zero (Student’s t-tests, *p* < 0.01). RII = (Bw – Bo) / (Bw + Bo), where Bw and Bo represent fungal abundance isolate in co- and mono-inoculation, respectively (Armas et al. 2004). RII > 0: facilitation, RII < 0: competition, RII = 0: neutral interaction

Regarding AMF competition, significant interactions of the factors fungal isolate and mycorrhizal stage (see Supplementary Table S6) confirmed that differences in the competitive response of AMF depended on mycorrhizal stage in both co-inoculation treatments (3 AMF: F_2, 25_ = 3.79, *p* < 0.05; 2 AMF: F_1, 18_ = 44.96, *p* < 0.0001). When all three AMF isolates were co-inoculated, each showed consistently negative RII values, indicating competition in both mycorrhizal stages. The competitive response of *R. irregularis* was less negative than the responses of the other two isolates at the early stage, suggesting asymmetric competition. In contrast, the competition was symmetric at the late stage, where the negative responses did not significantly differ among the three AMF. When only two AMF isolates were co-inoculated, both showed negative RII values at the early stage, indicating competition. While *E. claroidea* and *F. mosseae* had similar competitive responses initially, they diverged over time: at the late stage, only *E. claroidea* maintained a negative response, whereas *F. mosseae* increased in abundance compared to mono-inoculation, suggesting facilitation at this stage (Fig. 3).

### Abundance of the three AMF in different host plant species

**Fig. 4:**
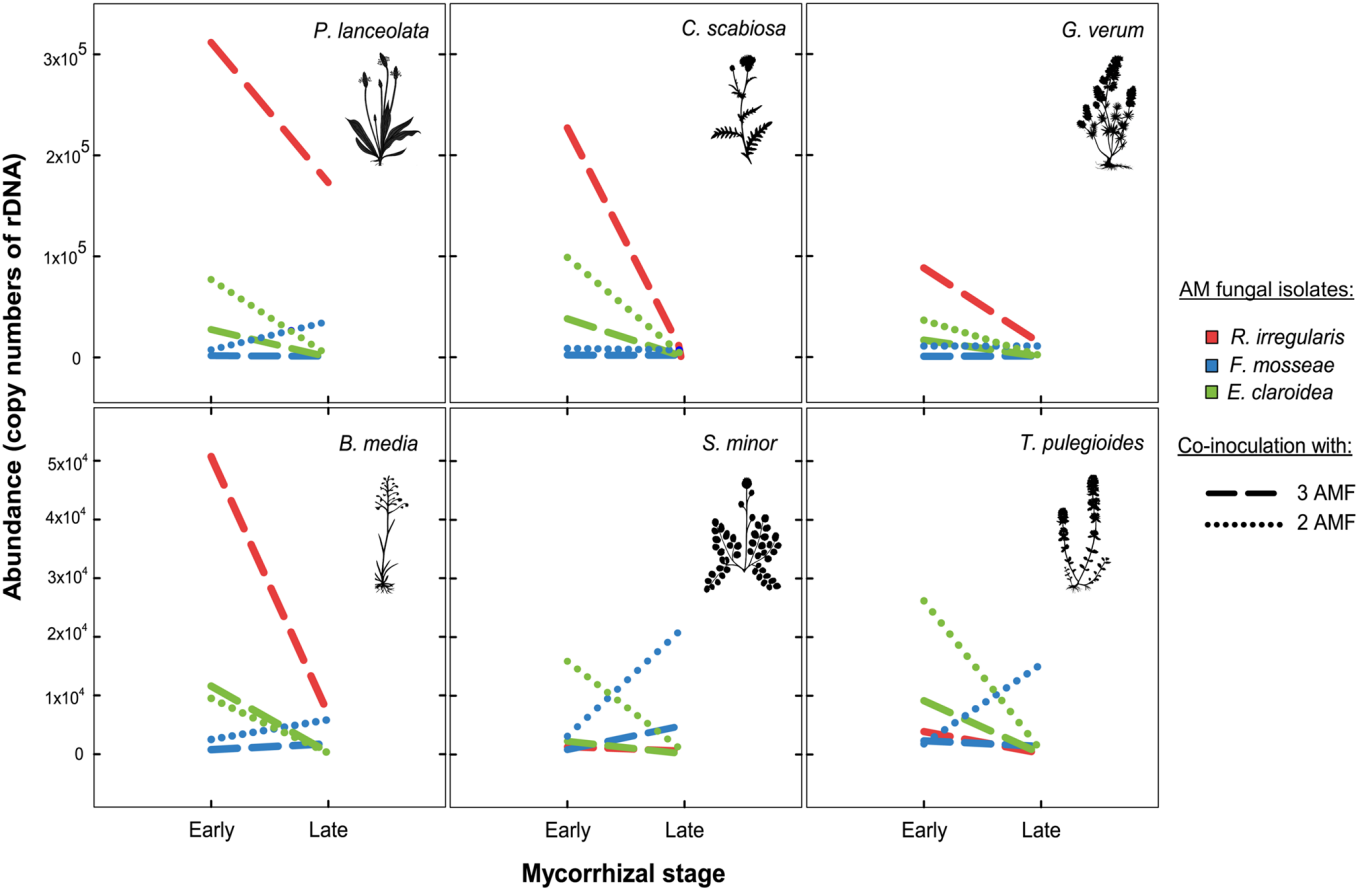
Abundance of arbuscular mycorrhizal fungi (AMF: *R. irregularis*, *F. mosseae*, and *E. claroidea*) co-occurring in six different host plant species: *P. lanceolata*, *C. scabiosa*, *G. verum*, *B. media*, *S. minor* and *T. pulegioides*. Values of abundance are showed per co-inoculation (3 or 2 AMF) during two mycorrhizal development stage (early and late). For standard error and significant differences between the means see Table 2

The effect of host plant species on the abundances of the fungal isolates depended on mycorrhizal stage in both co-inoculation treatments (significant interactions of both factors, 3 AMF: F_5, 159_ = 11.94, *p* < 0.0001; 2 AMF: F_5, 106_ = 15.90, *p* < 0.0001, see Supplementary Table S7). As compared to the host *P. lanceolata*, *R. irregularis* displayed a similar pattern of abundance in *C. scabiosa*, *G. verum* and *B. media* (Fig. 4). However, the decrease in the late stage was more pronounced in these three host plant species, to abundance levels comparable with the other two fungal isolates (Table 1). In contrast, all fungal isolates displayed comparable abundance levels in *S. minor* and *T. pulegioides*. In the treatment with 3 AMF co-inoculated, *E. claroidea* and *F. mosseae* also displayed a similar abundance pattern in *C. scabiosa*, *G. verum*, *B. media* and *T. pulegioides* as compared to *P. lanceolata* (Fig. 4, dashed lines). It consisted in decreasing abundance of *E. claroidea* between the early and late stage, and similar abundance of *F. mosseae* in both stages (Table 1). Differently, the abundance of *F. mosseae* increased in *S. minor* between the early and late stage. In the treatment with 2 AMF (Fig. 4, dotted lines), *E. claroidea* and *F. mosseae* displayed similar abundance patterns in *B. media*, *S. minor* and *T. pulegioides* as in *P. lanceolata*, i.e., abundance decrease in *E. claroidea* and increase in *F. mosseae* between the early and late stage (Table 1). In *C. scabiosa* and *G. verum*, the abundance of *F. mosseae* remained low in the late stage.

**Table 1:** Abundances of arbuscular mycorrhizal fungi (AMF: *R. irregularis*, *F. mosseae* and *E. claroidea*) in different host plant species (*P. lanceolata*, *C. scabiosa*, *G. verum*, *B. media*, *S. minor* and *T. pulegioides*) during two mycorrhizal development stages (early and late). Values are means ± standard error. Different letters indicate significant differences between the AMF isolates (3 AMF: a, b, c, d; 2 AMF: x, y, z) within each host plant species during both mycorrhizal stages (*a* *posteriori* *Tukey* *test*, *p* *<* *0.05*). *: only one replicate

Host	Stage	Abundance (copy number of rDNA)				
		Co-inoculation with 3 AMF			Co-inoculation with 2 AMF	
		*R. irregularis*	*F. mosseae*	*E. claroidea*	*F. mosseae*	*E. claroidea*
*P. lanceolata*	Early	311902 ± 36567a	1675 ± 624d	27619 ± 5315c	7565 ± 2363z	77103 ± 14641x
	Late	173041 ± 5841b	1276 ± 280d	487 ± 166d	35608 ± 6279y	2306 ± 767z
*C. scabiosa*	Early	227116 ± 42318a	2119 ± 894c	38067 ± 7153b	8822 ± 3379y	98916 ± 12580x
	Late	836 ± 305c	2058 ± 279c	151 ± 23c	7186 ± 1584y	233 ± 107z
*G. verum*	Early	883001 ± 10910a	758 ± 302c	16825 ± 1652b	10971 ± 3754y	36529 ± 2009x
	Late	13334 ± 6502b	1164 ± 308c	631 ± 117c	11111 ± 2374y	1276 ± 254z
*B. media*	Early	50659 ± 6182a	761 ± 350c	11599 ± 4099b	2499 ± 1049y	9496 ± 1355x
	Late	6780 ± 3756bc	1748 ± 233c	273 ± 76c	5879 ± 1109xy	177 ± 28z
*S. minor*	Early	1297 ± 549ab	808 ± 424bc	2265 ± 765ab	3103 ± 902y	15891 ± 2578x
	Late	568 ± 121c	4962 ± 1054a	61 ± 14c	21807 ± 5305x	385 ± 109z
*T. pulegioides*	Early	3889 ± 828ab	2321 ± 574ab	9171 ± 2700a	1813 ± 1240y	26201 ± 1053x
	Late	169 ± 121c	1375 ± 92b	99 ± 22c	15366 ± 0.00*x	645 ± 0.00*z

**Table 2:** Linear models of mycorrhizal growth response (MGR) as a function of the abundances of arbuscular mycorrhizal fungi (AMF: *R. irregularis*, *F. mosseae*, and *E. claroidea*) and host plant species during the mycorrhizal stages (early and late). Significant *p*-values are shown in bold. The individual contribution of each fungal predictor is given as partial R². ND: not determined, as the fungal predictor was non-significant according to the *p*-value

			MGR				
			Df	F-ratio	*p*-value	ß coefficient	Partial *R*^2^
		Abundance of *R. irregularis*	1	7.48	**0.0111**	-7.6 × 10^− 8^	0.0015
	early	Abundance of *F. mosseae*	1	8.45	**0.0074**	1.5 × 10^− 5^	0.0405
	stage	Abundance of *E. claroidea*	1	9.20	**0.0054**	2.9 × 10^− 6^	0.0654
Co-inoculation		Host plant species	5	10.63	**0.0000**		
with 3 AMF							
		Abundance of *R. irregularis*	1	3.02	0.0986	ND	ND
	late	Abundance of *F. mosseae*	1	4.54	0.0464	ND	ND
	stage	Abundance of *E. claroidea*	1	10.07	**0.0050**	-1.33 × 10^− 4^	0.1812
		Host plant species	5	4.16	**0.0101**		
	early	Abundance of *F. mosseae*	1	2.03	0.1658	ND	ND
	stage	Abundance of *E. claroidea*	1	65.03	**0.0000**	1.70 × 10^− 7^	0.0013
Co-inoculation		Host plant species	5	12.47	**0.0000**		
with 2 AMF							
	late	Abundance of *F. mosseae*	1	0.58	0.4539	ND	ND
	stage	Abundance of *E. claroidea*	1	0.31	0.5824	ND	ND
		Host plant species	5	5.61	**0.0016**		

## Mycorrhizal effect on plant growth

**Fig. 5:**
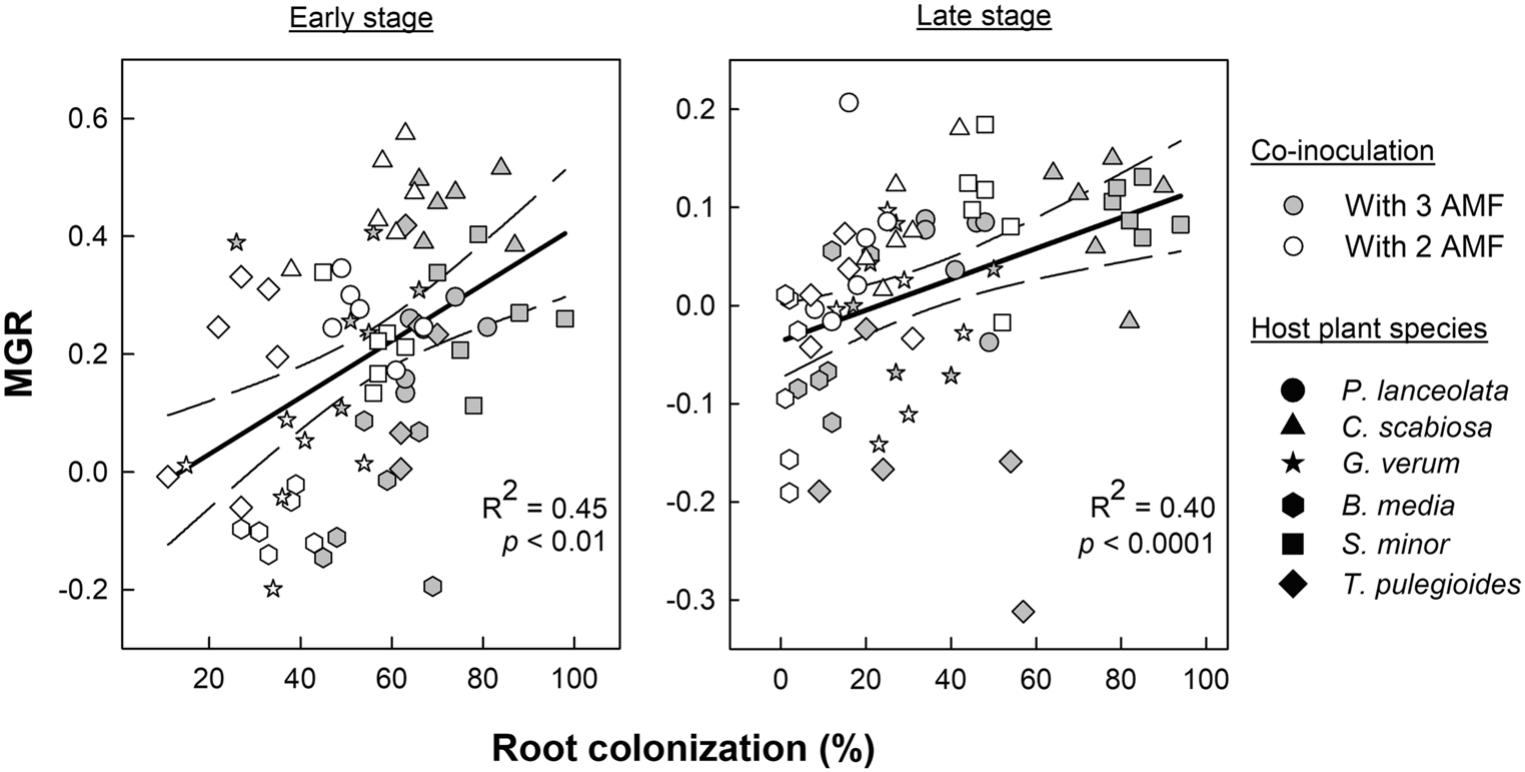
Relationship of mycorrhizal growth response (MGR) and percentage of root colonization at the early and late stages of mycorrhization. Data points represent treatments with either three (filled symbols) or two (open symbols) arbuscular mycorrhizal fungi (AMF) across different host plant species (*P. lanceolata*, *C. scabiosa*, *G. verum*, *B. media*, *S. minor*, and *T. pulegioides*). Solid lines represent the fitted linear regression models, with dashed lines indicating 95% confidence intervals. For mean values and standard error of both parameters see Supplementary Table S8

Across both co-inoculation treatments and all plant species, MGR displayed a significant positive relationship with RC at both stages (Fig. 5). In the treatment with three AMF co-inoculated, the abundances of all fungal isolates were significantly related to MGR at the early stage (Table 2). The abundances of *E. claroidea* and *F. mosseae* were positively related and contributing more to MGR variation than that of *R. irregularis*, which was negatively related to MGR. At the late stage, only *E. claroidea* abundance remained a significant predictor of MGR displaying a negative relationship. In the treatment with only two AMF co-inoculated, MGR was positively related to *E. claroidea* abundance at the early stage, while neither AMF isolates significantly contributed to MGR variation at the late stage. MGR significantly varied among the host plant species in all four models.

## Discussion

As expected, the relative abundances of the fungal isolates in both co-inoculations were broadly consistent with their growth rates in mono-inoculation in *P. lanceolata* (Fig. 2). *R. irregularis* consistently achieved the highest abundance and acted as a dominant colonizer that rapidly occupies root space, as previously observed in other systems (Deja-Sikora et al. 2023; Cook et al. 2024; Blažková et al. 2021). While the dominance of *R. irregularis* may partly reflect its higher vesicle production compared with the other isolates (INVAM, https://invam.ku.edu/), the high level of microscopically determined root colonization of this isolate in mono-inoculation (see Supplementary Table S8) supports the pattern established by the qPCR quantification. *E. claroidea* and *F. mosseae* were less extensive colonizers but also displayed distinct temporal dynamics: *E. claroidea* attained moderate abundance early but declined over time, whereas *F. mosseae* maintained more persistent colonization. These dynamics illustrate general life-history models for AMF (Hart and Reader 2002; Chagnon et al. 2013; Janoušková et al. 2013; Jansa et al. 2008): extensive colonizers, less extensive colonizers with early decline, and less extensive colonizers with persisting presence. While *E. claroidea* exhibited a consistent growth pattern regardless of competitor presence, the other two AMF partially altered their intraradical growth in co-inoculation compared with mono-inoculation: At the late stage, the abundance of *R. irregularis* declined and *F. mosseae* flexibly increased its abundance. Altogether, these results highlight the importance of considering fungal traits related to life-history strategies when interpreting AMF dynamics and competitive outcomes.

Having established that AMF abundances vary according to species-specific growth strategies, we next examined how these differences translated into their competitive responses. The less negative RII of *R. irregularis* at the early stage confirms its initial competitive advantage, consistent with its rapid colonization displayed in mono-inoculation in *P. lanceolata*. However, the convergence of the RII values among all isolates at the late stage indicates that competition became more symmetric as colonization progressed. These patterns are consistent with studies reporting transient asymmetry in AMF competition during early colonization (Engelmoer et al. 2014; Thonar et al. 2011), although progressive asymmetry with mycorrhizal development has also been observed (Voříšková et al. 2019). In the co-inoculation of two AMF only, the shift from symmetric to asymmetric competition between *E. claroidea* and *F. mosseae* may reflect the absence of suppression by *R. irregularis*, allowing the remaining fungi to access or partition limiting resources more effectively. While direct fungal-fungal competition cannot be ruled out during root colonization by multiple AMF, plant-mediated competition, i.e., for plant-derived C is a more plausible explanation (Pearson et al. 1994). Changes in host carbon allocation might as observed in other multi-symbiont systems (Bever et al. 2009; Kiers et al. 2011; Verbruggen et al. 2013), might contribute to competitive outcomes. Therefore, these results show that AMF competition is not static but context-dependent, modulated by both fungal traits and mycorrhiza development. The way how it is mediated by host plant traits and physiological status is clearly an important question for future research.

Our comparison of AMF species ratios between several plant species indeed supports that competitive traits of AMF are not inherent but partly depend on plant host species (Frew et al. 2023; Werner and Kiers 2015). The early dominance of *R. irregularis* in most of the tested plant species suggests that they provide sufficient carbon to support rapid spread of a ruderal fungus. In contrast, *S. minor* and *T. pulegioides* did not support this spread, either due to better control on the carbon flow to fungi or due to metabolic traits of these plant species, which generate less surplus carbon (Bunn et al. 2024). This is consistent with a role of host-specific factors, such as carbon allocation dynamics, in shaping fungal colonization (Bever et al. 2009). The two hosts, which did not support *R. irregularis* dominance, share few common traits beyond being, within our set of plant species, those with the lowest scores for R selection, whereas *P. lanceolata*, the species with the highest score, maintained *R. irregularis* dominance even at the later stage. As expected, (see Supplementary Table S2), *S. minor* and *T. pulegioides* appear to be generally less mycorrhiza-dependent (Table 1), and it may also limit colonization by other AMF species. Although preferential carbon allocation to more beneficial fungi has been proposed as a mechanism (Bennett and Bever 2009; Weber et al. 2024), evidence indicates that this process is complex and context-dependent, influenced by host nutrient demand, resource availability, and fungal responsiveness (Johnson et al. 2010; Kiers et al. 2011; Bever 2015). Alternating dominance between *E. claroidea* and *F. mosseae* across hosts further implies that variation in host quality and carbon dynamics can modulate fungal competition, reflecting an interplay between plant regulation and fungal responsiveness rather than direct penalization of less efficient symbionts (Grman 2012; Knegt et al. 2016).

Contrary to expectations, the most abundant or competitive AMF did not consistently provide the highest contribution to mycorrhizal growth response. This disconnect likely reflects fungal quality rather than quantity. AMF taxa may differ in nutrient supply, with some facilitating phosphorus uptake and others nitrogen, which may not match host demand (Smith and Smith 2015; Walder and van Der Heijden 2015). Highly abundant fungi can also impose high carbon costs with limited benefit (Kiers et al. 2011; Graham 2000), and variation in arbuscule formation may restrict nutrient transfer even when biomass is high (Bever et al. 2009; Lekberg and Koide 2005). In contrast, slower-growing fungi such as *E. claroidea* may better support host growth during early stage (Table 2). The disappearance of significant relationships of AMF abundance and mycorrhizal growth response of the host at the late stage may reflect declining fungal dominance as mycorrhizal networks equilibrate or as host demands change (Smith and Read 2010; Smith and Smith 2015). Overall, mycorrhizal benefits appear driven not solely by fungal abundance but by dynamic feedback between host carbon allocation and AMF resource-use strategy (Lekberg and Koide 2005; Bever et al. 2009; Weber et al. 2024).

The consistent positive relationship between MGR and RC (as measure of total fungal abundance in roots, Fig. 5) further indicates that the host benefit depends on overall fungal activity rather than on the abundance of any single AMF taxon (Werner et al. 2018). Nevertheless, recent studies report mixed evidence, indicating that colonization intensity alone does not always reliably predict mycorrhizal growth response (Bennett and Groten 2022; Horsch et al. 2023; Corrêa et al. 2024; Frew 2025). In addition to intraradical abundance, traits of the extraradical mycelium (ERM) may contribute to MGR. By extending the fungal absorptive surface into the soil, ERM influences nutrient foraging and host nutrient status, potentially shaping plant-mediated interactions (Smith and Read 2010; Antunes et al. 2025). Variation in ERM development and efficiency may therefore modulate overall fungal performance and plant responses (Dodd et al. 2000; Kokkoris 2026), highlighting an underexplored factor in plant-AMF interactions.

To sum up, our results demonstrate that AMF competition and host benefit are dynamic outcomes shaped by fungal life-history traits, host identity, and mycorrhizal stage. Fast-growing fungi such as *R. irregularis* dominated early colonization, but this advantage diminished as slower fungal isolates (i.e., *E. claroidea* and *F. mosseae*) persisted or recovered, revealing a shifting balance of competitive strategies. Host plant species further modulated these patterns, likely through differences in carbon allocation or compatibility (Kiers et al. 2011; Bennett and Bever 2009). These patterns suggest that plant functional strategies, potentially aligned with CSR axes, may interact with AMF life-history traits, but testing such trait matching will require experiments explicitly designed to quantify both plant and fungal functional traits. The most competitive fungus did not provide the greatest host benefit, as plant growth was more strongly linked to the total fungal abundance than to dominance by any fungal isolate. Therefore, maintaining a diversity of AMF functional types, rather than introducing single, highly competitive isolates, appears essential for stable and efficient plant-AMF associations in agricultural systems (Frew et al. 2023; van Der Heijden et al. 2015). Future studies should couple fungal abundance with physiological indicators of nutrient uptake by AMF and exchange with the host plant to clarify how complementarity and host regulation support mutualism stability and productivity under field conditions (Chagnon et al. 2013; Thonar et al. 2014; Bennett and Groten 2022).

## Supplementary Information

Below is the link to the electronic supplementary material.

Supplementary Material 1 (DOCX 60.8 KB)

## Data Availability

The data supporting the findings of this study are available in the Figshare repository at 10.6084/m9.figshare.31242232
