# Supplementary material for: Competitive dynamics of arbuscular mycorrhizal fungi as depending on fungal traits and host plant species
Source: Mycorrhiza. 2026 Feb 24;36(2):10. doi: 10.1007/s00572-026-01254-7 (PMC12929356; doi:10.1007/s00572-026-01254-7)
Supplement: Supplementary file 1 — Supplementary Material 1 (DOCX 60.8 KB) [file 572_2026_1254_MOESM1_ESM.docx]

**Online Resource 1 (Supplementary material)**

**Mycorrhiza**

**Competitive dynamics of arbuscular mycorrhizal fungi as depending on fungal traits and host plant species**

*Henry J. De La Cruz^1,2^*, Nicolás Marro^1,3^, Milena Caccia^1,3^, Kateřina Žďárská^1^, Martina Janoušková^1^*

1) Institute of Botany, The Czech Academy of Sciences, Zámek 1, 252 43 Průhonice, Czech

Republic

2) Department of Botany, Faculty of Science, Charles University, Benátská 2, 128 00, Prague, Czech Republic

3) [Instituto Multidisciplinario de Biología Vegetal (IMBIV), CONICET, Facultad de Ciencias Exactas, Físicas y Naturales, Universidad Nacional de Córdoba, Córdoba, Argentina](https://colab.ws/organizations/056tb7j80)

*Corresponding author, e-mail: [henry.delacruz@ibot.cas.cz](mailto:henry.delacruz@ibot.cas.cz)

**Table S1.** Previously documented life-history and competitive traits of the arbuscular mycorrhizal fungal (AMF) isolates used in this study, which help to contextualize their growth and colonization strategy.

| **AMF species** | **Key biological characteristics of AMF species*** | **AMF isolate** | **Colonization of the AMF isolates**** | **Competitive ability of the AMF isolates**** |
| --- | --- | --- | --- | --- |
| *Rhizophagus*  *irregularis* | Fungal model with variable spore morphology; many vesicles; frequent dense intra-radical clustering indicating high infectivity potential | PH5 | Fast colonizer, generally higher colonization compared to *F. mosseae* | Strong competitor in mixtures; commonly dominant |
| *Funneliformis*  *mosseae* | Spores often in clusters; wide geographic distribution and easily propagated in trap culture | BEG95 | Moderate colonizer; often lower colonization than  *R. irregularis* | Less competitive in mixtures compared to  *R. irregularis* |
| *Entrophospora*  *claroidea* | Species occurs widely in diverse soils, suggesting broad ecological tolerance | BEG23 | Moderate colonizer, similar to *F. mosseae* | Lower competitive dominance; often persists without dominating |

* International Collection of (Vesicular) Arbuscular Mycorrhizal Fungi (INVAM) - <https://invam.ku.edu/>

**Janoušková et al. 2013, Voříšková et al. 2019, Blažková et al. 2021

**Table S2.** CSR strategy, ecological description, mycorrhizal response (or root colonization) of the six host plant species. C: competitor, S: stress-tolerant, R: ruderal.

| **Plant Species** | **Family** | **CSR**  **strategy*** | **CSR Score Summary**  **(C:S:R %) *** | **Ecological description** | **Mycorrhizal response /**  **root colonization** | **References** |
| --- | --- | --- | --- | --- | --- | --- |
| *Plantago lanceolata L.* | Plantaginaceae | CR | C = 61.1 %, S = 0.9 %, R = 38 % | Broadly adapted competitor–ruderal; common in managed grasslands | High | Pawlowska et al. 1997; Romero et al. 2023 |
| *Centaurea scabiosa L.* | Asteraceae | C | C = 57 %, S = 15 %, R = 28 % | Competitive perennial herb of mesotrophic grasslands | Moderate to high | Pánková et al. 2018; Moora et al. 2004 |
| *Galium verum L.* | Rubiaceae | CS | C = 1.3 %, S = 71.8 %, R = 26.9 % | Stress-tolerant perennial herb of nutrient-poor grasslands | Moderate to high | Romero et al. 2023; Pánková et al. 2018 |
| *Briza media L.* | Poaceae | SR | C = 18.6 %, S = 49.5 %, R = 31.9 % | Common in semi-natural grasslands; moderate competitiveness and persistence under stress. | Moderate | Pánková et al. 2018; Dixon 2002 |
| *Sanguisorba minor Scop.* | Rosaceae | CS | C = 35.8 %, S = 41.1 %, R = 23.1 % | Perennial herb of calcareous grasslands; intermediate competitor and stress-tolerator | Moderate | Pánková et al. 2018; Gay et al. 1982 |
| *Thymus pulegioides L.* | Lamiaceae | S | C = 0 %, S = 76.8 %, R = 23.2 % | Dwarf perennial adapted to dry, nutrient-poor soils | Low to moderate | Pawlowska et al. 1997 |

Approximate qualitative range based on published compilations of mycorrhizal response / root colonization across different plant species.

*Pladias – Database of the Czech Flora and Vegetation. www.pladias.cz

**Table S3.** Real-time PCR assays and reaction conditions used for the quantification of the three fungal isolates, based on primers and hydrolysis probes targeted to the large subunit of nuclear ribosomal DNA (nrLSU) (Thonar et al. 2012; Voríšková et al. 2017, 2019). **E. claroidea* (formerly known as *Glomus claroideum* or *Claroideoglomus claroideum*).

|  |  |  | **Primer** | **Annealing** | **Size of** |
| --- | --- | --- | --- | --- | --- |
| **target isolate** | **type** | **sequence 5'-3'** | **conc.** | **Temperature** | **Amplicon** |
|  |  |  | **(nM)** | **(°C)** | **(bp)** |
| *Rhizophagus irregularis PH5* | primerF | TTCGGGTAATCAGCCTTTCG | 400 | 58 | 249 |
|  | primerR | TCAGAGATCAGACAGGTAGCC |  |  |  |
| *Funneliformis mosseae BEG95* | primerF | GGAAACGATTGAAGTCAGTCATACCAA | 400 | 58 | 121 |
|  | primerR | CGAAAAAGTACACCAAGAGATCCCAAT |  |  |  |
| *Entrophospora claroidea BEG23** | primerF | GCGAGTGAAGAGGGAAGAG | 400 | 52 | 178 |
|  | primerR | TTGAAAGCGTATCGTAGATGAAC |  |  |  |

**Table S4.** Schematic diagram of composition of a) the substrate in each pot, and b) the inoculum per treatment (control, mono-inoculation and co-inoculation). RI: *R. irregularis*, FM: *F. mosseae*, EC: *E. claroidea*.

a)


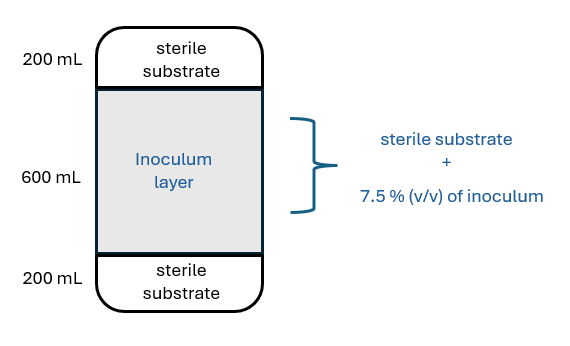


b)

| **Inoculum** | control | Mono-inoculation | | | Co-inoculation | |
| --- | --- | --- | --- | --- | --- | --- |
| **% (v/v)** | **Non-AMF** | **RI** | **FM** | **EC** | **FM + EC** | **RI + FM + EC** |
| **Living** | 0 | 0 | 2.5 | 2.5 | 2.5 + 2.5 | 2.5 +2.5 +2.5 |
| **Blanked** | 7.5 | 7.5 | 5 | 5 | 2.5 | 0 |
| **total** | 7.5 | 7.5 | 7.5 | 7.5 | 7.5 | 7.5 |

**Table S5.** Summary of two-way ANOVAs used to determine the effect of the factors fungal isolate, mycorrhizal stage and their interaction on the fungal abundance (as copy numbers of rDNA) in the roots of *P. lanceolata*. The analyses were separately performed and are shown per each inoculation treatments: mono-inoculation, co-inoculation with 3 or 2 arbuscular mycorrhizal fungi (AMF). Significant *p*-values are shown in bold.

|  | **Abundance** | | |
| --- | --- | --- | --- |
|  | **Df** | **F-ratio** | ***p*-value** |
| **Mono-inoculation** |  |  |  |
| Fungal isolate (F) | 2 | 25.44 | **6.1 x 10^-7^** |
| Mycorrhizal stage (S) | 1 | 1.85 | 0.1853 |
| F x S | 2 | 7.17 | **0.0032** |
| Residuals | 27 |  |  |
|  |  |  |  |
| **Co-inoculation with 3 AMF** |  |  |  |
| Fungal isolate (F) | 2 | 263.65 | **1.3 x 10^-15^** |
| Mycorrhizal stage (S) | 1 | 58.68 | **1.6 x 10^-7^** |
| F x S | 2 | 37.29 | **1.2 x 10^-7^** |
| Residuals | 21 |  |  |
|  |  |  |  |
| **Co-inoculation with 2 AMF** |  |  |  |
| Fungal isolate (F) | 1 | 0.74 | 0.4002 |
| Mycorrhizal stage (S) | 1 | 13.04 | **0.0017** |
| F x S | 1 | 95.78 | **4.5 x 10^-9^** |
| Residuals | 20 |  |  |

**Table S6.** Summary of two-way ANOVAs used to determine the effect of the factors fungal isolates, mycorrhizal stage and their interaction on the fungal response to competition (RII) in the roots of *P. lanceolata*. The analyses were separately performed and are shown per each co-inoculation treatment: co-inoculation with 3 or 2 arbuscular mycorrhizal fungi (AMF). Significant *p*-values are shown in bold.

|  | **RII** | | |
| --- | --- | --- | --- |
|  | **Df** | **F-ratio** | ***p*-value** |
|  |  |  |  |
| **Co-inoculation with 3 AMF** |  |  |  |
| Fungal isolate (F) | 2 | 8.39 | **0.0016** |
| Mycorrhizal stage (S) | 1 | 4.13 | 0.0528 |
| F x S | 2 | 3.79 | **0.0364** |
| Residuals | 25 |  |  |
|  |  |  |  |
| **Co-inoculation with 2 AMF** |  |  |  |
| Fungal isolate (F) | 1 | 17.84 | **0.0005** |
| Mycorrhizal stage (S) | 1 | 24.49 | **0.0001** |
| F x S | 1 | 44.96 | **0.0000** |
| Residuals | 18 |  |  |

**Table S7.** Summary of three-way ANOVAs used to determine the effect of the factors: fungal isolates, host plant identity, mycorrhizal stage and their interactions on the fungal abundance (as copy numbers of rDNA in roots), in co-inoculation with 3 or 2 arbuscular mycorrhizal fungi (AMF). Significant *p*-values are shown in bold.

|  | **Abundance** | | |
| --- | --- | --- | --- |
|  | **Df** | **F-ratio** | ***p*-value** |
|  |  |  |  |
| **Co-inoculation with 3 AMF** |  |  |  |
| Fungal isolate (F) | 2 | 89.51 | **< 2.2 x 10^-16^** |
| Host plant species (H) | 5 | 40.60 | **< 2.2 x 10^-16^** |
| Mycorrhizal stage (S) | 1 | 229.45 | **< 2.2 x 10^-16^** |
| F x H | 10 | 18.11 | **< 2.2 x 10^-16^** |
| F x S | 2 | 132.01 | **< 2.2 x 10^-16^** |
| H x S | 5 | 11.94 | **8.1 x 10^-10^** |
| F x H x S | 10 | 3.40 | **0.0005** |
| Residuals | 159 |  |  |
|  |  |  |  |
| **Co-inoculation with 2 AMF** |  |  |  |
| Fungal isolate (F) | 1 | 17.37 | **6.29 x 10^-5^** |
| Host plant species (H) | 5 | 24.34 | **3.03 x 10^-16^** |
| Mycorrhizal stage (S) | 1 | 129.72 | **< 2.2 x 10^-16^** |
| F x H | 5 | 9.92 | **8.36 x 10^-8^** |
| F x S | 1 | 405.72 | **< 2.2 x 10^-16^** |
| H x S | 5 | 15.90 | **1.15 x 10^-11^** |
| F x H x S | 5 | 10.72 | **2.35 x 10^-8^** |
| Residuals | 106 |  |  |

**Table S8.** Mycorrhizal growth response (MGR) and percentage of root colonization (RC) in the different host plant species (*P. lanceolata*, *C. scabiosa*, *G. verum*, *B. media*, *S. minor* and *T. pulegioides*). Mean values are shown per inoculation treatment: mono-inoculation, co-inoculation with 3 or 2 arbuscular mycorrhizal fungi (AMF), and during two mycorrhizal development stages (early and late). *: Value significantly different from zero (Student’s t-tests).

|  |  | **Host** | **MGR** | | | **RC** | | |
| --- | --- | --- | --- | --- | --- | --- | --- | --- |
|  |  |  |  |  |  |  |  |  |
|  | Mono-inoculation |  |  |  |  |  |  |  |
|  | *R. irregularis* |  | 0.29 | **±** | 0.04* | 69.33 | **±** | 2.06 |
|  | *F. mosseae* | *P. lanceolata* | -0.12 | **±** | 0.03* | 10.17 | **±** | 3.30 |
|  | *E. claroidea* |  | 0.26 | **±** | 0.01* | 45.17 | **±** | 1.49 |
|  |  |  |  |  |  |  |  |  |
|  |  | *P. lanceolata* | 0.22 | **±** | 0.03* | 68.67 | **±** | 3.00 |
|  |  | *C. scabiosa* | 0.45 | **±** | 0.02* | 74.67 | **±** | 3.63 |
|  | Co-inoculation | *G. verum* | 0.28 | **±** | 0.05* | 50.50 | **±** | 5.46 |
|  | with 3 AMF | *B. media* | -0.05 | **±** | 0.05 | 56.83 | **±** | 3.93 |
|  |  | *S. minor* | 0.27 | **±** | 0.04* | 81.33 | **±** | 4.11 |
| Early |  | *T. pulegioides* | 0.19 | **±** | 0.07 | 64.60 | **±** | 1.54 |
| stage |  |  |  |  |  |  |  |  |
|  |  | *P. lanceolata* | 0.26 | **±** | 0.02* | 54.67 | **±** | 3.16 |
|  |  | *C. scabiosa* | 0.46 | **±** | 0.03* | 57.00 | **±** | 3.99 |
|  | Co-inoculation | *G. verum* | -0.01 | **±** | 0.04 | 36.17 | **±** | 5.15 |
|  | with 2 AMF | *B. media* | -0.09 | **±** | 0.02* | 35.17 | **±** | 2.40 |
|  |  | *S. minor* | 0.22 | **±** | 0.03* | 56.17 | **±** | 2.46 |
|  |  | *T. pulegioides* | 0.17 | **±** | 0.07 | 25.83 | **±** | 3.53 |
|  |  |  |  |  |  |  |  |  |
|  | Mono-inoculation |  |  |  |  |  |  |  |
|  | *R. irregularis* |  | 0.29 | **±** | 0.04* | 69.33 | **±** | 2.06 |
|  | *F. mosseae* | *P. lanceolata* | -0.12 | **±** | 0.03* | 10.17 | **±** | 3.30 |
|  | *E. claroidea* |  | 0.26 | **±** | 0.01* | 45.17 | **±** | 1.49 |
|  |  |  |  |  |  |  |  |  |
|  |  | *P. lanceolata* | 0.06 | **±** | 0.02* | 42.00 | **±** | 2.77 |
|  |  | *C. scabiosa* | 0.09 | **±** | 0.03* | 76.33 | **±** | 3.74 |
|  | Co-inoculation | *G. verum* | 0.00 | **±** | 0.03 | 30.33 | **±** | 5.06 |
|  | with 3 AMF | *B. media* | -0.04 | **±** | 0.03 | 11.50 | **±** | 2.26 |
|  |  | *S. minor* | 0.10 | **±** | 0.01* | 83.83 | **±** | 2.36 |
| Late |  | *T. pulegioides* | -0.17 | **±** | 0.05* | 32.80 | **±** | 9.60 |
| Stage |  |  |  |  |  |  |  |  |
|  |  | *P. lanceolata* | 0.06 | **±** | 0.03 | 16.50 | **±** | 2.45 |
|  |  | *C. scabiosa* | 0.08 | **±** | 0.02* | 28.50 | **±** | 3.08 |
|  | Co-inoculation | *G. verum* | -0.03 | **±** | 0.04 | 27.17 | **±** | 4.02 |
|  | with 2 AMF | *B. media* | -0.08 | **±** | 0.04 | 2.00 | **±** | 0.45 |
|  |  | *S. minor* | 0.10 | **±** | 0.03* | 48.50 | **±** | 1.59 |
|  |  | *T. pulegioides* | 0.01 | **±** | 0.02 | 15.20 | **±** | 4.39 |

**References**

Blažková A, Jansa J, Püschel D, Vosátka M, Janoušková M (2021) Is mycorrhiza functioning influenced by the quantitative composition of the mycorrhizal fungal community? Soil Biology and Biochemistry 157: 108249.

Dixon JM (2002) *Briza media* L. Journal of Ecology 90(4): 737-752.

Gay PE, Grubb PJ, Hudson HJ (1982) Seasonal changes in the concentrations of nitrogen, phosphorus and potassium, and in the density of mycorrhiza, in biennial and matrix-forming perennial species of closed chalkland turf. The Journal of Ecology 571-593.

Grime JP (1988) The CSR model of primary plant strategies-origins, implications and tests. In Plant evolutionary biology 371-393.

Janoušková M, Krak K, Wagg C, Štorchová H, Caklová P, Vosátka M (2013) Effects of inoculum additions in the presence of a preestablished arbuscular mycorrhizal fungal community. Applied and environmental microbiology 79(20): 6507-6515.

Moora M, Öpik M, Zobel M (2004) Performance of two Centaurea species in response to different root-associated microbial communities and to alterations in nutrient availability. In Annales Botanici Fennici 263-271.

Pánková H, Lepinay C, Rydlová J, Voříšková A, Janoušková M, Dostálek T, Münzbergová Z (2018) Arbuscular mycorrhizal fungi and associated microbial communities from dry grassland do not improve plant growth on abandoned field soil. Oecologia 186(3): 677-689.

Pawlowska TE, Błaszkowski J, Rühling Å (1997) The mycorrhizal status of plants colonizing a calamine spoil mound in southern Poland. Mycorrhiza 6(6): 499-505.

Romero F, Argüello A, de Bruin S, van Der Heijden MG (2023) The plant-mycorrhizal fungi collaboration gradient depends on plant functional group. Functional Ecology 37(9): 2386-2398.

Thonar C, Erb A, Jansa J (2012) Real-time PCR to quantify composition of arbuscular mycorrhizal fungal communities--marker design, verification, calibration and field validation. Molecular Ecology 12: 219-232.

Voríšková A, Jansa J, Püschel D, Krüger M, Cajthaml T, Vosátka M, Janoušková M (2017) Realtime PCR quantification of arbuscular mycorrhizal fungi: does the use of nuclear or mitochondrial markers make a difference? Mycorrhiza 27: 577-585.

Voříšková A, Jansa J, Püschel D, Vosátka M, Šmilauer P, Janoušková M (2019) Abiotic contexts consistently influence mycorrhiza functioning independently of the composition of synthetic arbuscular mycorrhizal fungal communities. Mycorrhiza 29(2): 127-139.
